# Mis-reporting, previous health status and health status of family may seriously bias the association between food patterns and disease

**DOI:** 10.1186/1475-2891-9-48

**Published:** 2010-10-30

**Authors:** Agneta Hörnell, Anna Winkvist, Göran Hallmans, Lars Weinehall, Ingegerd Johansson

**Affiliations:** 1 Department of Food and Nutrition, Umeå University, Umeå, Sweden; 2 Department of Clinical Nutrition, Sahlgrenska Academy, University of Gothenburg, Gothenburg, Sweden; 3 Nutrition Research, Department of Public Health and Clinical Medicine, Umeå University, Umeå, Sweden; 4 Epidemiology and Global Health, Department of Public Health and Clinical Medicine, Umeå University, Umeå, Sweden; 5 Cariology, Department of Odontology, Umeå University, Umeå, Sweden

## Abstract

**Background:**

Food pattern analyses are popular tools in the study of associations between diet and health. However, there is a need for further evaluation of this methodology. The aim of the present cross-sectional study was to evaluate the relationship between food pattern groups (FPG) and existing health, and to identify factors influencing this relationship.

**Methods:**

The inhabitants of Västerbotten County in northern Sweden are invited to health check-ups when they turn 30, 40, 50, and 60 years of age. The present study includes data collected from almost 60,000 individuals between 1992 and 2005. Associations between FPG (established using K-means cluster analyses) and health were analyzed separately in men and women.

**Results:**

The health status of the participants and their close family and reporting accuracy differed significantly between men and women and among FPG. Crude regression analyses, with the high fat FPG as reference, showed increased risks for several health outcomes for all other FPGs in both sexes. However, when limiting analysis to individuals without previous ill-health and with adequate energy intake reports, most of the risks instead showed a trend towards protective effects.

**Conclusions:**

Food pattern classifications reflect both eating habits and other own and family health related factors, a finding important to remember and to adjust for before singling out the diet as a primary cause for present and future health problems. Appropriate exclusions are suggested to avoid biases and attenuated associations in nutrition epidemiology.

## Background

Food pattern analyses have become popular in recent decades for the study of associations between diet and health, especially regarding prospective studies on all-cause mortality and cardiovascular disease (CVD) [1,2]. One reason for the popularity of this strategy is that it emphasizes the entire diet as opposed to single nutrients or foods.

Accurate food intake information is needed for unbiased estimates of the impact of diet, including food patterns, on the etiology and progression of different diseases. Correct adjustments for individual characteristics likely related to both food patterns and later health outcomes (confounding factors) are required. Furthermore, dietary intake and measurement of health outcomes should be separated in time, i.e. assessed prospectively in epidemiological studies. Otherwise, reversed causality may occur in that progression of disease may be associated with selection of a certain dietary pattern in the hope of curing disease.

To study the effect of confounding and reversed causality on identification of associations between diet and future health outcomes, a large source of data is needed where information exists to classify individuals into groups with or without estimated bias. Currently, there are a limited number of large, population-based studies of diet and health that would allow for this analysis. One such study is the on-going Västerbotten Intervention Programme (VIP). In the VIP, food intake was recorded by a food frequency questionnaire that has been validated against interview-obtained data and laboratory biomarkers [3-5].

In a previous publication we reported on food patterns identified through cluster analysis, as well as macro- and micronutrient intake in women and men taking part in the VIP [6]. The aim of the present study was to assess the relationship between food patterns and existing health problems, and to identify factors that might be influential on these relationships and contribute to bias in studies of disease etiology.

## Methods

### Study base

Since 1985, as a part of a community intervention programme for the prevention of CVD and diabetes, the inhabitants of Västerbotten County in northern Sweden (total population approximately 255,000) have been invited by their local health center to take part in the VIP when they turn 40, 50, or 60 years of age. In the early years of the program, 30-year-olds were also included. Yearly participation rates vary between 57-66%. No systematic differences between participants and non-participants were found in a sample from 1992-1993 [7]. The present population-based cross-sectional study includes data collected between 1992 and 2005 from the participants' first visit to the VIP. Data collected before 1992 were not optically readable and therefore not included. The food intake measurements, validation, subjects, and food pattern analysis are described briefly below; more details are given in our previous publication [6].

### Food intake measurements

Individual food intake is reported through a semi-quantitative food frequency questionnaire (FFQ) covering the preceding 12-month period. Between 1992 and 1996, the FFQ included 84 food items, such as edible fats, fruits, vegetables, milk and milk products, bread, potatoes, rice, pasta, fish, meat and meat products, chicken, traditional dishes, hot and cold beverages, sweets, sugar and jam, and snacks. From 1996 this was reduced to 66 food items by deleting entire foods (e.g. liver and kidney) or by merging similar foods(e.g. merging the two groups 'apples, pears, peaches' and 'oranges, mandarines, grape fruit' into one group 'apples, pears, peaches, oranges, mandarines, grape fruit'). The two data sources have been harmonized and combined into one file for the purpose of the food pattern analysis.

Portion sizes for the three categories of potato/rice/pasta, meat/fish, and vegetables are indicated by participants through comparison with color photos of four plates with increasing portion sizes. Frequency of dietary intake is reported on a 9-level scale from none to ≥ 4 times daily. For the analysis, these frequencies were transformed to a daily frequency.

Daily intake of energy and nutrients was calculated by multiplying frequency of intake by a portion size value and by the energy and nutrient content found in the food composition database from the Swedish National Food Administration [8]. Portion sizes used were those indicated on the photos, natural sizes such as an orange, or average portion sizes for sex and age [3]. Energy and nutrient contents were calculated using the software MATs (Rudans Lättdata, Sweden). Macronutrient intake is reported both as absolute intake in gram/day and as nutrient density, i.e. proportion of total energy, E%.

The 84-item FFQ has previously been validated in a sub-sample using ten repeated 24-h recalls, two repeated FFQs, and the biomarkers plasma β-carotene, erythrocyte fatty acids, and plasma B vitamins [3-5]. The FFQ was deemed valid for the ranking of individuals on their dietary intake.

To assess levels of low energy reporting the individual's reported food intake level (FIL) was compared with his or her estimated physical activity level (PAL), based on reported physical activity level at work and at leisure [9]. FIL was calculated by dividing reported total caloric intake with estimated basal metabolic rate (BMR) [10], and PAL was calculated by dividing estimated total energy expenditure with BMR [11].

"Low energy reporting" defined as a calculated energy intake <PAL × 0.748 (the lower 95% CI limit of FIL in the VIP population), was a common phenomenon among both women (59.1%) and men (60.4%). In accordance with other studies, low energy reporting was more common among those with higher body mass index (BMI, kg/m^2^), increased age, and lower education. "High energy reporting" defined as a calculated energy intake >PAL × 1,336 (the upper 95% CI limit of the FIL), was rare among both women (0.3%) and men (0.1%), and they were included in the group Adequate Reporters.

### Subjects

Food intake patterns were evaluated for 62,531 individuals (32,600 women and 29,931 men) based on food data from their first health check-up within the VIP. Only individuals with acceptable data on portion sizes, intake frequencies, and FIL were included in the analysis. In total, 1,347 men and 1,245 women were excluded due to missing information on portion sizes and/or missing consumption frequencies on more than 10% of the food items. For more than half of those excluded the reason was missing portion size and for almost half missing frequencies. About 300 lacked information on both portion size and frequencies. In addition, individuals with a FIL below the 5^th ^percentile or above the 97.5^th ^were excluded (n = 5,425).

Due to the large sample size, most differences between included and excluded individuals showed large statistical significance. Important differences were healthier life style habits, lower BMI, a higher frequency of being married, higher education, and lower rate of smoking among those included in the analyses.

For the present study, 1,596 women and 1,256 men were excluded due to missing data on physical activity and, as BMI was one of the independent variables in the regression analysis, an additional 50 women and 56 men were excluded due to implausible height and/or weight (for women; <130 cm, >200 cm, or <30 kg, for men <140 cm, >215 cm, or <40 kg). Thus the final sample included 59,573 individuals (30,954 women and 28,619 men).

### Food pattern analysis

Cluster analysis was used to evaluate food intake patterns for men and women separately. SPSS, version 14 (Chicago, IL, USA) was used for the analyses (Quick Cluster procedure, K-means method). Food items/aggregates listed in the FFQ were in the end grouped into 36 meaningful food groups according to nutrient content (especially fat quantity and quality, and fibre content) and/or culturally relevant culinary preferences.

To increase robustness in the cluster analysis several steps were taken; 1) energy adjustment was used by calculating frequencies of intake per 1000 kcal; 2) differences among the clusters in intake of all included food groups were scrutinized and the F-statistic for the analysis of variance tables was inspected by AH and AW, first separately and then by comparing evaluations; 3) emerging patterns were evaluated for two to ten clusters for both sexes; 4) five random variables were used to sort the data in both ascending and descending order before the analysis, and cluster assignment was robust for both sexes across the ten runs obtained; 5) to further test the robustness, the cluster analyses were repeated after removing the low energy reporters, and also repeated separately on the 64-item and the 84-item FFQs. The emerging patterns remained stable for both sexes.

Four food pattern groups (FPG) were identified for women: *High fat *(high intake of high-fat spreads and high-fat milk products), *Tea & ice cream *(high intake of tea, ice cream), *Coffee & sandwich *(high intake of coffee, low-fat spreads, cold cuts), and *Fruit & vegetables *(high intake of fruit, vegetables, chicken, fish, red meat, boiled potato).

Three FPGs were identified for men: *High fat *(high intake of high-fat spreads and milk products, coffee, fat in cooking, sugar & jam, beer), *Tea, soda, & cookies *(high intake of tea, soda, cookies), and *Fruit & vegetables *(high intake of fruit, vegetables, high fiber bread, low-fat spreads and milk products, chicken, cold cuts, wine, fried potatoes).

Overall, women reported a larger variation in intake than men, which was reflected in the number of FPGs established for each sex. In addition, for women mean intakes were similar across FPGs for two of the 36 food groups compared with ten food groups for the men [6]. The women also had a larger SD in mean frequency of daily intake for most food groups.

### Health measurements and lifestyle questionnaire

#### Biomedical factors

The health check-up included measurements of weight and height, glucose load, blood lipids [serum cholesterol (S-chol), serum triglycerides (S-TG), serum high density lipoprotein (S-HDL), serum low density lipoprotein (S-LDL)], blood pressure, smoking and snuff use, and alcohol intake. Details about the data collection procedure are published elsewhere [12].

For the present study, the guidelines of the Swedish Medical Products Agency for prevention of cardiovascular disease were used [13]. S-lipids were regarded as increased if S-TG ≥ 1.7 mmol/L and/or S-HDL < 1.3 mmol/L for women and/or < 1.0 mmol/L for men. Blood pressure was regarded as elevated if systolic pressure was ≥ 140 mmHg (≥ 130 if diabetic) and/or diastolic pressure was ≥ 90 mmHg (≥ 80 if diabetic), and/or a person took medication for elevated blood pressure. Capillary blood was used for the glucose tolerance test. A person was regarded as having impaired fasting glucose (IFG) if fasting S-glucose level was 6.1-6.9 mmol/L and 2 hr S-glucose was < 8.9 mmol/L, impaired glucose tolerance (IGT) if fasting S-glucose level was < 7.0 mmol/L and 2 hr S-glucose level was 8.9-12.1 mmol/L, and diabetic if fasting S-glucose level was ≥ 7.0 mmol/L and/or 2 hour S-glucose level ≥ 12.2 mmol/L.

The questionnaire also included questions about the participants' current medication and previously diagnosed medical conditions (high blood pressure, diabetes, stroke, myocardial infarction). A dichotomous variable *Previously Ill *(yes/no) was constructed and included medication for elevated S-lipids or high blood pressure, and having diabetes, stroke or myocardial infarction prior to the health exam. Participants who stated that a doctor or nurse had ever told them that they had high blood pressure, but who did not take medication for this, were not considered previously ill (12.7% of women and 9.5% of men). These participants and all others without any previously known health problems are, for the sake of simplicity, called Previously Healthy in the remaining text. One hundred and thirty-four individuals (0.2% of each FPG), lacked information about previous health.

'Perceived health' was assessed by one question "How do you judge your overall health status during the last year?". The questionnaire also included questions about the health of parents and siblings and a dichotomous variable *Health of parents and siblings *(ill/healthy) was constructed including high blood pressure, diabetes, and stroke.

#### Social variables

For the present study, answers to the lifestyle questionnaire were grouped into the following categories: education (≤ 9 years, 10-12 years, university), living in urban area (population centre with more than 15,000 inhabitants, yes/no), cohabiting status (together with adult with or without children/only with children/living alone), activity level at leisure (< 1/week, 1/week, >1/week), smoking (never or occasional smoker/ex-smoker/present smoker), snuff use (never or occasional user/ex-user/present user), alcohol use (not problematic/problematic). For alcohol use to be deemed problematic the participant should have answered *yes *on at least one of the following three questions: "Have others irritated you by criticizing your drinking?"; "Have you ever felt bad or had feelings of guilt about your drinking habits?"; or "Have you ever taken a drink first thing in the morning to calm your nerves or to recover from a hangover"?

### Statistical measures

Differences in background factors between FPG among women and men were evaluated via ANOVA and Chi-square test, while differences in nutrient intakes were investigated using Kruskal-Wallis analysis of variance test and Chi-square test.

Associations between FPG and background factors, nutrient intakes, and results of the health check-up were performed on the whole sample as well as separately for the four groups: (i) Previously Healthy Adequate Reporters (9,376 women, 8,691 men), (ii) Previously Healthy Low Energy Reporters (14,300 women, 14,061 men), (iii) Previously Ill Adequate Reporters (2,311 women, 1,862 men), and (iv) Previously Ill Low Energy Reporters (4,895 women, 3,943 men). Most differences among FPG for Previously Healthy were significant regardless of reporting accuracy, while most differences among FPG for Previously Ill were non-significant. However, in almost all analyses the order with respect to macro- and micronutrient intake between the FPGs within the sexes was identical regardless of subgroup.

Multivariate logistic regression analyses were performed with FPG as predictor of five dichotomized medical health outcomes: (i) elevated S-lipids (yes/no), (ii) elevated blood pressure (yes/no), (iii) diabetes (yes/no), (iv) IGT or diabetes (yes/no), and (v) IFG or IGT or diabetes (yes/no). The *High fat *groups were used as reference for both women and men. Multivariate ordinal regression was used to assess FPG as a predictor of perceived health.

All regression analysis were initially stratified by age group (30, 40, 50, 60 years old) and adjusted for BMI, education, cohabiting status, size of the local municipality, and lifestyle (physical leisure activity, smoking, snuff and alcohol use). For all independent, categorical variables a category *missing *was added when data were incomplete. As the relation between FPG and health outcomes did not differ between age groups, final analysis were carried out on all age groups combined, adjusted for age. The variables *Previously Ill *(ill/healthy) as well as *Health of parents and siblings *(ill/healthy) were also included as covariates in the final regression analysis. SPSS, version 17 (Chicago, IL, USA) was used for the regression analyses.

The project was approved by the regional ethical committee, Gothenburg, Sweden.

## Results

Overall the proportion of individuals per FPG varied according to reporting accuracy (*P *< 0.001 for both sexes, Table 1). The proportion of individuals classified as *Fruit & vegetables *was higher among Low Energy Reporters than among Adequate Reporters for both sexes. The proportions of women classified as *Tea & ice cream *and men classified as *Tea, soda, & cookies *were higher among Adequate Reporters than Low Energy Reporters.

**Table 1:** Reporting accuracy in relation to food pattern group and sex (the Västerbotten Intervention Program 1992-2005).^1^

	Women				Men		
**Food pattern group (FPG), women (n)**	**Total** **(30,954)**	**Adequate Reporters** **(11,712)**	**Low Energy Reporters** **(19,242)**	**Food pattern group, (FPG) men (n)**	**Total** **(28,619)**	**Adequate Reporters** **(10,570)**	**Low energy Reporters** **(18,049)**

High fat	32.9^2^	35.3	30.8	High fat	29.1	20.2	34.3
(10,061)	100^3^	41.1	58.9	(8,331)	100	25.6	74.4
Tea & ice cream	25.1	32.8	20.4	Tea, soda, & cookies	40.2	53.6	32.4
(7,770)	100	49.4	50.6	(11,517)	100	49.2	50.8
Coffee & sandwich	28.0	23.1	30.9	Fruit & vegetables	30.7	26.2	33.2
(8,663)	100	31.3	68.7	(8,771)	100	31.6	68.4
Fruit & vegetables	14.4	8.7	17.9				
(4,460)	100	22.9	77.1				
Total	100	100	100	Total	100	100	100
(30,954)	100	37.8	62.2	(28,619)	100	36.9	63.1

^1 ^Chi-square test used to test differences within sex. All differences are significant at *P *< 0.001 for both sexes.

^2^Top number = proportion in relation to reporting accuracy (Total, Adequate Reporters, Low Energy Reporters).

^3^Bottom number = proportion within FPG.

Health problems of the participants themselves or of their close family (i.e, parents or siblings), diagnosed before the health check-up, differed significantly between women and men and among FPGs, respectively (Table 2). Overall, men had significantly more known health problems than women, but stated their relatives to be healthier than those of the women. For both sexes, *Fruit & vegetables *had the largest proportion of participants with previously known health problems and the largest proportions of health problems among family.

**Table 2:** Known health problems prior to health check-up in relation to food pattern group and sex (the Västerbotten Intervention Program 1992-2005).^1-3^

Sex	Women					Men			
	
Food pattern group (FPG) **(n)**^**4**^	Total (30,882)	High fat (10,033)	Tea & ice cream (7,755)	Coffee & sandwich (8,643)	Fruit & vegetables (4,451)	Total (28,557)	High fat (8,311)	Tea, soda, & cookies (11,494)	Fruit & vegetables (8,752)
Previous participant health problems (total)^5^	10.3	8.8	8.1	11.9	**14.5**	10.8	9.2	8.6	**15.4**
Previous diabetes	0.9	0.6	0.6	1.1	**1.8**	1.6	1.1	1.1	**2.6**
Previous S-lipid medication	1.0	0.6	0.7	1.5	**1.8**	2.4	1.8	1.5	**4.1**
Previous blood pressure medication	9.2	8.1	7.2	0.7	**12.5**	8.4	7.2	6.9	**11.6**
Previous stroke	0.3	0.3	0.2	**0.4**	0.3	1.5	1.3	1.0	**2.2**
Health problems in family^6, 7^	36.2	35.2	32.8	38.3	**40.3**	32.5	33.1	30.1	**35.2**
Diabetes in family^6^	21.2	20.1	18.8	23.0	**24.6**	17.6	17.5	16.1	**19.6**
Cardiac infarction or stroke in family^7^	20.6	20.3	18.6	21.6	**23.1**	19.0	19.7	17.7	**20.1**

^1^Chi-square test used to test differences among FPGs within sex. All differences are significant at *P *< 0.001, except "Previous stroke" among women where *P *= 0.122.

^2 ^Numbers given are percentages.

^3 ^Largest proportion within sex in bold.

^4 ^In total, 134 individuals (0.2% of each FPG) lacked information about previous health and are therefore not included in the table.

^5 ^Diabetes, medication for elevated S-lipid levels and/or hypertension, and stroke prior to health check-up.

^6 ^Diabetes in parents or siblings.

^7^Cardiac infarction or stroke prior to 60 years of age in parents or siblings.

To avoid bias introduced by the different proportions of participants with previously known health problems and low energy reporters among the FPGs, all analyses were repeated in the different subgroups (as stated in Methods), and only results for the subgroup of Previously Healthy Adequate Reporters are reported in the remaining text.

Among Previously Healthy Adequate Reporters, all background factors differed significantly between the sexes (*P *< 0.001 for all variables) and most of them differed between FPGs within sex (*P *< 0.01 for most variables) (Table 3). Problematic alcohol use and snuff use were more common among men than women (four and five times higher, respectively), and men were almost twice as likely to be overweight. The female and male *Fruit & Vegetables *groups were oldest, had the highest BMI, were more likely to live in larger cities, and were among those most likely to exercise frequently. The female *Tea & ice cream *group and the male *Tea, soda, & cookies *group were the youngest, more often single, least likely to smoke or use snuff, and also had a high level of physical activity. The female and male *High Fat *groups were more likely to live in smaller cities, to smoke and use snuff, to have problems with alcohol, and least likely to do regular physical activity.

**Table 3:** Background characteristics in relation to food pattern group and sex among Previously Healthy Adequate Reporters (the Västerbotten Intervention Program 1992-2005).^1, 2^

Sex	Women					Men			
	
Food pattern group (FPG)(n)	Total (9,376)	High fat (3,398)	Tea & ice cream (3,122)	Coffe & sandwich (2,100)	Fruit & vegetables (756)	Total (8,691)	High fat (1,790)	Tea, soda, & cookies (4,726)	Fruit & vegetables (2,175)
Age, (years)	44.5 ± 9.0	45.2 ± 8.8	42.0 ± 9.1	46.0 ± 8.4	47.4 ± 8.4	46.0 ± 9.5	46.8 ± 8.7	45.6 ± 10.0	46.5 ± 9.0
Age group:									
30 y	15.7	12.1	26.2	9.6	5.9	13.5	8.2	16.6	11.1
40 y	37.6	39.7	36.8	36.2	35.0	33.4	35.4	33.3	32.1
50 y	34.0	33.3	29.1	40.3	38.8	33.2	37.7	29.1	38.4
60 y	12.8	14.9	7.9	13.9	20.3	19.9	18.7	21.0	18.4
Height (m)	165.0 ± 5.9	164.8 ± 5.9	165.5 ± 6.1	164.8 ± 5.7	164.5 ± 5.9	178.4 ± 6.6	177.6 ± 6.4	178.7 ± 6.7	178.6 ± 6.6
Weight (kg)	65.3 ± 10.5	64.4 ± 9.8	65.3 ± 10.5	66.1 ± 11.1	67.5 ± 10.9	80.1 ± 11.0	78.3 ± 10.3	80.5 ± 11.2	80.7 ± 11.1
BMI (kg/m^2^)	24.0 ± 3.6	23.7 ± 3.4	23.9 ± 3.7	24.3 ± 3.9	24.9 ± 3.8	25.1 ± 3.1	24.8 ± 2.9	25.2 ± 3.1	25.3 ± 3.0
BMI group:									
≤ 24.99	67.9	70.5	69.2	65.5	58.5	51.8	56.0	51.5	49.1
25.0-29.99	25.4	24.3	24.6	26.2	31.0	41.6	39.1	41.4	44.0
30.0-34.99	5.5	4.4	4.9	6.7	8.5	5.9	4.6	6.3	6.0
≥ 35	1.2	0.8	1.3	1.6	1.9	0.7	0.3	0.7	0.8
Cohabiting status:									
w/adult (± child)	86.4	86.3	85.4	88.0	87.0	86.8	87.6	86.1	87.6
only w/child	6.4	6.4	6.8	5.9	5.8	2.3	2.7	2.1	2.3
single	7.0	7.1	7.6	5.9	6.9	10.6	9.3	11.4	9.8
Urban area^3^	66.6	62.5	68.3	69.1	71.4	74.0	59.0	63.0	70.1
Education:									
≤ 9 ys	19.0	23.3	12.8	22.0	17.7	22.5	28.7	21.6	19.8
10-12 yrs	47.5	48.7	46.2	50.4	42.7	53.3	53.6	53.4	53.8
university	33.0	28.0	41.0	27.6	39.6	23.7	17.8	24.9	26.5
Smoking:									
smoker	19.0	26.4	10.0	23.2	14.2	15.7	27.5	12.1	14.6
ex-smoker	16.8	16.9	14.3	19.8	19.6	20.9	23.8	19.3	23.4
non-smoker	63.4	56.8	75.7	56.9	66.2	62.0	48.7	68.6	62.9
Snuff use:									
snuff user	5.2	5.7	4.3	4.7	4.9	26.9	32.7	24.2	23.8
ex-user	4.3	4.8	3.8	3.3	3.7	15.2	15.5	13.5	16.4
non-user	90.5	84.1	86.7	85.8	84.4	57.8	47.6	58.0	56.6
Problematic alcohol use	7.1	8.3	6.8	6.4	5.1	21.1	22.3	21.4	19.5
Physical leisure activity:									
< 1/week	74.0	78.7	68.9	76.6	67.5	76.7	86.1	73.7	75.6
1/week	18.5	16.2	21.2	17.6	20.3	13.9	9.9	15.4	14.0
> 1/week	7.4	5.1	9.9	5.8	12.2	9.3	4.0	10.8	10.4

^1 ^ANOVA and Chi-square test used to test differences between the sexes and between FPGs within sex. All differences between sexes significant at *P *< 0.001. All differences between FPGs within sex are significant at *P *< 0.01, except for "Cohabiting status" among women where *P *= 0.26 and "Cohabiting status" and "Problematic alcohol use" among men where *P *= 0.09 and *P *= 0.08, respectively.

^2 ^Numbers given are mean ± SD or percentages.

^3 ^Population centre more than 15,000 inhabitants.

Among the Previously Healthy Adequate Reporters, in general, larger variations in nutrient intake were seen among female FPGs than male FPGs, although all differences among FPGs within each sex were significant (see Additional file 1, Table S1-S4). The *High fat *group of both sexes reported the lowest intake of protein, carbohydrates, and fiber as well as most vitamins and minerals. Among women, the *High fat *group also had the highest intake of fat, saturated fatty acids, and cholesterol, and the lowest intake of polyunsaturated fatty acids. Consistently, the *Fruit & vegetables *group represented the opposite end of the spectrum. Among men, the *High fat *group reported the highest fat density intake (E%), but the *Tea, soda, & cookies *group reported a higher absolute fat intake (g/day) and the highest intake of saturated fatty acids and cholesterol. Although significantly different, the reported density intake of protein, sugar, and alcohol were similar among FPGs within each sex. In women, the intake of fiber, carotenoids, vitamin C, folate, and saturated fatty acids showed the largest differences among FPGs.

In Previously Healthy Adequate Reporters, more health problems were found at the health check-up among men than among women (*P *< 0.001 for all variables). About one-fourth of the men and one-fifth of the women had elevated S-lipid levels, with the largest proportion in the *High fat *groups for both sexes (Table 4). Elevated blood pressure was almost twice as common among men as women, with the largest proportion in the *Fruit & vegetables *group for both sexes, although not significantly different for men. IFG, IGT, and diabetes were similar between FPGs among both sexes.

**Table 4:** Result at the health check up among Previously Healthy Adequate Reporters (the Västerbotten Intervention Program 1992-2005).^1^

Sex	Women					Men			
	
Food pattern group (FPG) (n)	Total (9,376)	High fat (3,398)	Tea & ice cream (3,122)	Coffe & sandwich (2,100)	Fruit & vegetables (756)	Total (8,691)	High fat (1,790)	Tea, soda, & cookies (4,726)	Fruit & vegetables (2,175)
Diagnoses at health checkup (%)									
Elevated S-lipid levels	18.1	19.6	16.4	18.7	17.3	22.8	25.7	22.8	20.6
Elevated blood pressure	9.2	9.1	7.8	10.5	11.8	15.9	16.1	15.6	16.4
Diabetes	1.3	1.5	1.2	1.2	1.6	2.1	2.2	2.0	2.4
IGT	4.7	4.8	4.8	4.6	4.6	3.3	2.4	3.7	3.2
IFG	7.3	8.0	6.6	7.0	7.2	10.8	11.4	10.5	10.9
									
Perceived health (%)									
bad/relatively bad	6.6	6.6	7.4	5.8	6.2	4.7	5.0	4.9	4.0
fair	20.1	20.6	20.2	20.0	19.4	19.9	22.4	19.5	19.1
relatively good	47.2	47.8	46.9	48.6	45.5	49.9	49.0	50.7	49.6
good	25.5	25.0	25.5	25.7	28.9	25.1	23.6	24.9	27.3

Abbreviations: IGT = Impaired Glucose Tolerance; IFG = Impaired Fasting Glucose

^1^ANOVA and Chi-square test used to test differences between sexes and among FPGs within sex. All differences between sexes significant at *P *< 0.001. Among female FPGs differences in "S-lipid levels" and "Blood pressure" are significant at *P *< 0.01; remaining differences non-significant ("Diabetes" *P *= 0.55, "IGT" *P *= 0.97, "IFG" *P *= 0.17, "Perceived health" *P *= 0.17). Among male FPGs differences in "S-lipid levels" and "Perceived health" are significant at *P *< 0.01, "IGT" *P *= 0.03; remaining differences non-significant ("Blood pressure" *P *= 0.65, "Diabetes" *P *= 0.54, and "IFG" *P *= 0.49).

**Figure 1:**
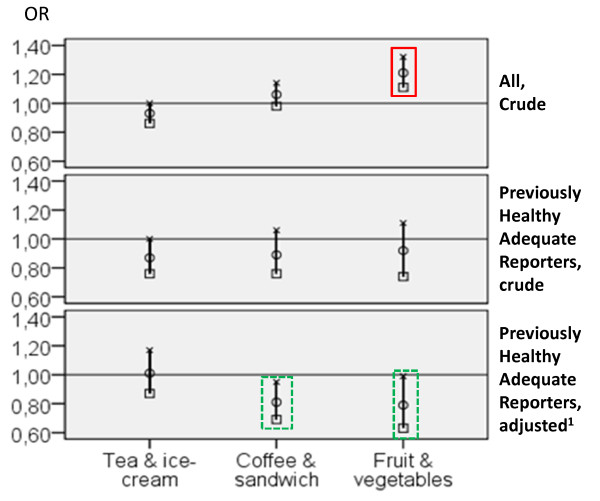
**Relation between food patterns groups (FPGs) among women and risk of being diagnosed with IFG/IGT/diabetes**. Multivariate regression analysis of relation between food patterns groups (FPGs) and risk of being diagnosed with IFG/IGT/diabetes at the health check-up among women in the whole population (crude - top), and among only the Previously Healthy Adequate Reporters (crude - middle and adjusted - bottom), in northern Sweden, 1992-2005. (OR and 95% CI). "High fat" FPG used as reference group. Abbreviations: BMI = body mass index, kg/m^2^; CI = confidence interval; IFG = impaired fasting glucose; IGT = impaired glucose tolerance; OR = odds ratio; Significantly increased risk marked with red (solid line) and significantly decreased risk with green (dashed line). ^1^Adjusted for previously being told about high blood pressure (but not taking medication), family history of ill health, age, BMI, education, cohabiting status, living area, physical leisure activity, smoking, snuff use, alcohol use.

The relationship between FPG and risk of receiving a diagnosis at the health check-up was further evaluated in multivariate logistic regression analyses. In crude analysis of the whole population (before discriminating on reporting accuracy and previous health), significantly increased risks for elevated blood pressure, diabetes, and IGT/diabetes were found for the *Fruit & vegetables *groups of both sexes and for the female *Coffee & sandwich *group compared with the *High fat *groups (Table 5). For the *Fruit & vegetables *groups, the risk for IFG/IGT/diabetes was also increased. However, when the regression analysis were repeated (crude and adjusted) only among Previously Healthy Adequate Reporters, different results emerged. Most of the risks for the other FPGs, compared with the *High fat *group, instead showed a trend towards protective effects (see Figure 1 for illustration of the diagnosis IFG/IGT/diabetes). For the diagnosis of elevated S-lipid levels, most FPGs were associated with a decreased risk compared with the *High fat *groups, although in the final adjustment this was only significant for the male *Fruit & vegetables *group. The female *Tea & ice cream *and the male *Tea, soda, & cookies *groups were associated with a decreased risk for most health outcomes in the crude models.

**Table 5:** Multivariate analysis of relation between food pattern group and diagnoses at the health check-up (the Västerbotten Intervention Program 1992-2005).^1, 2^

Sex		Women			Men	
		
Food pattern group (FPG) (n)	High fat	Tea & ice cream (3,122)	Coffee & sandwich (2,100)	Fruit & vegetables (756)	Tea, soda, & cookies (4,726)	Fruit & vegetables (2,175)
**Elevated S-lipid levels**						
All, crude model^3^	1.0 (-)	0.86 (0.80-0.92)	1.02 (0.96-1.09)	0.92 (0.84-1.00)	0.87 (0.82-0.92)	0.96 (0.90-1.03)
Previously Healthy						
Adequate Reporters, crude	1.0 (-)	0.80 (0.71-0.91)	0.94 (0.83-1.07)	0.86 (0.71-1.04)	0.86 (0.76-0.96)	0.75 (0.65-0.87)
Previously Healthy						
Adequate Reporters, adjusted^4^	1.0 (-)	1.00 (0.88-1.14)	0.94 (0.82-1.08)	0.89 (0.72-1.09)	0.91 (0.80-1.04)	0.8 (0.68-0.93)
**Elevated blood pressure**						
All, crude model^3^	1.0 (-)	0.78 (0.71-0.85)	1.12 (1.04-1.21)	1.25 (1.14-1.38)	0.92 (0.86-0.98)	1.14 (1.06-1.22)
Previously Healthy						
Adequate Reporters, crude	1.0 (-)	0.85 (0.72-1.00)	1.17 (0.99-1.39)	1.34 (1.06-1.68)	0.97 (0.84-1.12)	1.03 (0.87-1.21)
Previously Healthy						
Adequate Reporters, adjusted^4^	1.0 (-)	1.03 (0.86-1.24)	1.02 (0.85-1.23)	1.02 (0.78-1.32)	0.89 (0.76-1.05)	0.94 (0.78-1.13)
**Diabetes**						
All, crude model^3^	1.0 (-)	0.99 (0.82-1.20)	1.28 (1.07-1.53)	1.67 (1.37-2.03)	0.98 (0.85-1.14)	1.48 (1.28-1.71)
Previously Healthy						
Adequate Reporters, crude	1.0 (-)	0.90 (0.56-1.43)	0.73 (0.42-1.28)	1.33 (0.69-2.55)	0.82 (0.56-1.22)	1.06 (0.69-1.63)
Previously Healthy						
Adequate Reporters, adjusted^4^	1.0 (-)	0.99 (0.6-1.62)	0.65 (0.37-1.15)	1.13 (0.58-2.21)	0.88 (0.59-1.33)	1.12 (0.71-1.75)
**IGT/diabetes**						
All, crude model^3^	1.0 (-)	1.05 (0.95-1.16)	1.13 (1.02-1.25)	1.27 (1.13-1.43)	1.10 (0.99-1.22)	1.34 (1.20 1.49)
Previously Healthy						
Adequate Reporters, crude	1.0 (-)	0.94 (0.77-1.14)	0.91 (0.73-1.13)	0.98 (0.72-1.33)	1.24 (0.97-1.59)	1.23 (0.93-1.63)
Previously Healthy						
Adequate Reporters, adjusted^4^	1.0 (-)	1.09 (0.89-1.34)	0.84 (0.67-1.05)	0.87 (0.64-1.20)	1.276 (0.98-1.64)	1.22 (0.92-1.63)
**IFG/IGT/diabetes**						
All, crude model^3^	1.0 (-)	0.93 (0.86-1.00)	1.06 (0.98-1.14)	1.21 (1.11-1.32)	0.93 (0.87-0.99)	1.09 (1.01 (1.17)
Previously Healthy						
Adequate Reporters, crude	1.0 (-)	0.87 (0.76-1.00)	0.89 (0.76-1.06)	0.92 (0.74-1.11)	0.98 (0.85-1.13)	1.01 (0.86-1.19)
Previously Healthy						
Adequate Reporters, adjusted^4^	1.0 (-)	1.01 (0.87-1.17)	0.81 (0.69-0.95)	0.79 (0.63-0.99)	1.06 (0.91-1.23)	1.04 (0.88-1.23)

Abbreviations: IGT = Impaired Glucose Tolerance; IFG = Impaired Fasting Glucose.

^1 ^The FPG High Fat used as comparison group among both women and men (n = 3,398 women, 1,790 men).

^2 ^Numbers given are odds ratio (95% confidence interval)

^3 ^All participants, before exclusion of previously ill participants and low energy reporters.

^4 ^Adjusted for previously being told about high blood pressure (without being given medication for it), family history of ill health, age, BMI, education, cohabiting status (with adult/living with only children/living alone), physical leisure activity (< 1/w, 1/w, >1/w), smoking (never, ex-smoker, present smoker), snuff use (never, ex-user, present user), alcohol use (not problematic/problematic).

Regarding perceived health, the initial Chi-2 test showed a significant difference between male FPGs but not female FPGs (Table 4). After adjustment for cofactors in the multivariate ordinal regression, no significant differences remained for either sex (data not shown).

## Discussion

The size of the VIP dietary database, with over 60,000 records between 1992 and 2005, is a significant strength of this study and provided the opportunity to evaluate relationships between food patterns and health among different subgroups with reasonable power. The level of low energy reporting as well as previously known health problems in the participants and their close relatives were taken into account, all of which are factors that likely confound the relationship between food intake and health. Due to the large number of participants, fairly small differences were usually highly significant at *P *< 0.01. As the aim of the current study was to identify factors that might contribute to bias in studies of disease etiology, using a cross-sectional design when looking at risk-markers for CVD (rather than prospectively studying CVD as an end-point) it can only generate hypotheses, and does not allow any conclusions on causality.

With data driven methods, such as cluster and factor analysis of food patterns, researchers are compelled to make numerous subjective decisions, and no gold standard exist for these procedures. Therefore, the usefulness of food patterns has been questioned [14,15], and the need for further development and evaluation of the methods is reported [1,16].

A problematic aspect of studies continuing over extended time periods is that it is plausible that data collection, end-point ascertainment as well as lifestyle habits vary during the data collection period. This is an important aspect, and an example of a two edged sword in nutritional epidemiology. On the one hand FFQ questions should be left unchanged for comparison, but on the other hand the panorama of food items change and with that selection preferences. We have not adjusted for screening year in the presented analyses. However, it is unlikely that end-point ascertainments have changed over time due to the general nature of these questions and standardization/calibration procedures for lab analyses, but lifestyle habits certainly have changed both in Sweden as a whole and in the study area. We are presently describing 25-year time trends in food selection in VIP, and in several parts the results accord with longitudinal production and consumption information (Johansson et al., manuscript). We mean that adoption of the questions to changes in the market is important when specific food components are to be evaluated, such as specific fatty acids, but that the generalized nature of the FFQ questions is sufficient for more general aspects, such as time trends and, as here, food patterns.

Another major concern is that many studies on dietary patterns do not validate their food intake data. The golden standard, doubly labeled water technique, is expensive and therefore not feasible in most trials, particularly in larger epidemiological studies. However, a comparison between reported energy intake and calculated energy expenditure should be a minimal requirement for all dietary intake studies, including food pattern studies [11]. This would enable exclusion of participants clearly misreporting their intake, as well as allowing for reporting the proportion of low energy reporters in each FPG. Mattisson et al [17] compared three common methods to classify misreporting in large-scale epidemiological studies and concluded that using individual PAL-values is preferable to using a fixed cut-off point. In the present study an individual PAL-value was calculated by comparing the reported physical activity at work and at leisure with the categories used in a two-question questionnaire on physical activity developed by Johansson and Westerterp [9]. This questionnaire has been validated with doubly labeled water in a small scale study with promising results, and seems to be suitable for use in large-scale epidemiological studies. Participants with implausible food intake data were excluded from the cluster analysis. After clusters had been developed, Low Energy Reporters were identified. The first un-adjusted analyses showed that the *Fruit & vegetables *group among women and the *High Fat *group among men reported the lowest energy intake. However, when the analyses were repeated with Adequate Reporters and Low Energy Reporters in separate groups, we found that the differences in energy intake in the crude analyses were explained by differences in the proportion of Low Energy Reporters in the FPGs, indicating that this is an important factor to recognize and discuss in relation to FPG results. Some studies report one, often very large, FPG with low energy intake [18,19]; however, authors often do not discuss whether this might be due to a higher level of low energy reporting in this group and what effect this might have on their conclusions. This failure to recognize and control for low energy reporting is one possible explanation for conflicting and/or inconsistent results when studying associations between food patterns and health. In the present study, the association with health was studied both for the total sample and separately for Adequate and Low Energy Reporters.

There are well known associations between food habits and characteristics such as gender, age, education, and socio-economic status, and with health behaviors such as physical activity, smoking, and drinking [1]. In the present study, we also found clear differences among FPG with regard to previous health of the participants and their close relatives. Some, but not all, studies on the relationship between food patterns and health have taken the participants previous medical history into account through exclusion, stratification, or adjustment [20], but we could not find any study that included the health of close relatives in the analysis. This may lead to remaining bias in estimates of the association between food patterns and later health.

The *Fruit & vegetables *groups of both sexes, the female *Tea & ice cream*, and the male *Tea, soda, & cookies *group reported the healthiest food choices [6]. Past food habit changes has been found to be more common in clusters with healthier food choices [21], and illness in family and friends has been shown to influence food choices [22]. Unfortunately, the participants were not asked about changes in food pattern, but in light of the background characteristics of these four groups, it might be possible that the latter two groups had a healthier lifestyle by choice whereas the lifestyle and food pattern of the former two might be more influenced by known health issues in themselves or close relatives. Increased knowledge about the reasons behind people's lifestyle choices and factors that can help or hinder healthy choices is important when planning health interventions and warrant further studies.

Interestingly, the latter two groups also had the highest proportion of Adequate Reporters whereas the female *Fruit & vegetables *group had the highest and the male *Fruit & vegetables *group the second highest proportion of Low Energy Reporters. This is in accordance with other studies showing that low energy reporting is associated with reporting a healthy food pattern [21,23]. Surprisingly, among males the *High fat *group had the highest level of low energy reporting.

Participants that did not take blood pressure medication but previously had been told by a doctor or nurse that they had high blood pressure were not classified as Previously Ill. This situation was more common among women, possibly reflecting the fact that many women have transient elevated blood pressure during pregnancy. However, it has been shown that untreated hypertension is associated with a four-fold increased risk for later stroke [24]. Thus, we adjusted for this in the final regression analysis as it may have influenced choices of lifestyle and food patterns.

Perceived health has been associated with the risk of CVD [12,25]. In the present study we found a significant association between FPG and perceived health for men only in the crude analysis (with highest proportion of participants with good health among the *Fruit & Vegetables *FPG), but this disappeared after adjustment.

We previously reported that most of the differences between women and men in food intake among VIP participants are consistent with Swedish national data [6,26]. Larger differences in nutrient intake, especially fat (E%), carbohydrate (E%), fiber (g), carotenoids, vitamin C, and folate, were seen among female FPG than among male FPG. In both sexes, *High fat *and *Fruit & vegetables *FPG represented the opposite ends of the intake continuum for most nutrients. In a review of cluster and factor analysis [1], Newby and Tucker point out that gender is an important factor to include in the analysis; either by deriving the food patterns separately for women and men or by including sex in further analysis after deriving food patterns in a mixed population. In the present study we found large differences between women and men, both with regard to food intake and relevant background factors, and therefore we believe that, if cluster analysis is performed on a mixed population, it would be useful if the gender distribution is given for each FPG. Newby and Tucker also point out that in many studies that separated women and men, the derived patterns were similar between sexes [1]. In the present study, the naming of the FPGs was similar between the sexes, even if actual food intake differed [6]. For instance, women belonging to the female *Fruit & vegetables *FPG ate fruit and vegetables on average three times more frequently than the men belonging to the male *Fruit & vegetables *FPG, who even ate less fruit and vegetables than the female FPG with the lowest frequency. These kinds of differences between FPGs with the same or similar names are important to consider when drawing conclusions about associations between food patterns and health.

Low energy reporting has been associated with BMI and desire of weight change [27,28], as well as with changed food pattern [21]. In the very first regression analysis, before discrimination between reporting accuracy and previous health, the associations found between FPG and health outcomes were contradictory to present recommendations in that the *Fruit & vegetables *FPG seemed to have the highest risk for many clinical diagnoses. However, the regression analyses were thereafter repeated on the Previously Healthy Adequate Reporters alone, to avoid reverse causality as individuals with previous health problems may choose to eat healthier. In these analyses, most of the risks for clinical outcomes associated with belonging to the *Fruit & vegetables *group were attenuated and this FPG instead became protective. In the Malmö Diet and Cancer Cohort it was found that past food habit change was related to obesity, lifestyle and socio-economic factors which can seriously distort observed relationships between diet and health [29]. They have also showed that exclusion of low energy reporters affects the results when studying associations between food patterns and cancer [17]. This highlights that it is of crucial importance to recognize that mis-reporting, as well as changed food habits due to previous ill health or other causes, are major threats to the validity of all nutrition epidemiology studies as it causes misclassification of dietary exposures, contributing to attenuated associations between food intake and health outcomes.

In the present study, being in the *High fat *groups was associated with an increased risk for adverse health effects, mainly for IFG/IGT/diabetes in females and elevated S-lipid levels in males. The *High fat *groups had the highest or second highest intake of most macro- and micronutrients that are viewed as unhealthy and the lowest intake of most nutrients viewed as healthy. The associations between food habits and health are complex and are further complicated by associations with weight and physical activity. It has been shown that being normal weight but physically inactive is more detrimental to long term health than being moderately overweight and physically active [30,31]. Whether a high intake of unhealthy foods or a low intake of healthy foods is most detrimental to long-term health is unclear. From a public health point of view a broad approach is necessary when planning health interventions.

## Conclusions

Food pattern classifications are associated with reporting accuracy and, in addition, reflect both eating habits and other own and family health related factors, a finding important to remember and to adjust for before singling out the diet as a primary cause for present and future health problems. Appropriate exclusions are suggested to avoid biases and attenuated associations in nutrition epidemiology.

## Abbreviations

BMI: body mass index (kg/m^2^); BMR: basal metabolic rate; CI: confidence interval; CVD: cardiovascular disease; E%: energy percentage; FIL: food intake level (calculated by dividing reported total caloric intake with estimated BMR); FFQ: food frequency questionnaire; FPG: food pattern group; IFG: impaired fasting glucose; IGT: impaired glucose tolerance; PAL: physical activity level (calculated by dividing estimated total energy expenditure with BMR); OR: odds ratio; S-CHOL: serum cholesterol; S-HDL: serum high density lipoprotein; S-LDL: serum low density lipoprotein; S-TG: serum triglycerides; VIP: Västerbotten Intervention Program

## Competing interests

The authors declare that they have no competing interests.

## Authors' contributions

GH, BL, LW, and IJ designed and carried out the Västerbotten Intervention Project (VIP) and the collection of dietary data. IJ was responsible for the validation of dietary data. AW and IJ were responsible for the creation of a coherent dietary data base. AH and AW carried out the statistical analysis. AH drafted the manuscript. All authors read and approved the final manuscript.

## Supplementary Material

Additional file 1 **Macro- and micronutrient intake among women (Tables S1 and S3) and men (Table S2 and S4) classified as Previously Healthy Adequate Reporters (the Västerbotten Intervention Program, 1992-2005)**. As supplementary information we present four tables on intake of macro- and micronutrients among Previously Healthy Adequate Reporters women and men. Many nutrients exhibit a skewed distribution; hence, both means ± standard deviations (SDs), and medians (25^th ^- 75^th ^percentiles) are included. Click here for file
