# Supplementary material for: Mis-reporting, previous health status and health status of family may seriously bias the association between food patterns and disease
Source: Nutr J. 2010 Oct 30;9:48. doi: 10.1186/1475-2891-9-48 (PMC2988699; doi:10.1186/1475-2891-9-48)
Supplement: Additional file 1 — Macro- and micronutrient intake among women (Tables S1 and S3) and men (Table S2 and S4) classified as Previously Healthy Adequate Reporters (the Västerbotten Intervention Program, 1992-2005). As supplementary information we present four tables on intake of macro- and micronutrients among Previously Healthy Adequate Reporters women and men. Many nutrients exhibit a skewed distribution; hence, both means ± standard deviations (SDs), and medians (25th - 75th percentiles) are included. [file 1475-2891-9-48-S1.DOCX]

# Additional file 1

## Additional file 1, Table S1. Macronutrient intake among women classified as Previously Healthy Adequate Reporters (the Västerbotten Intervention Program, 1992-2005)^1, 2^

| Food pattern group  (FPG) (n) | High fat (3,398) | Tea & ice-cream (3,122) | Coffee & sandwich (2,100) | Fruit & vegetables (756) |
| --- | --- | --- | --- | --- |
| Energy (kcal) | 2007 ± 314  1961 (1774-2205) | 2126 ± 350  2087 (1863-2350) | 1980 ± 298  1944 (1761-2148) | 2007 ± 296  1968 (1786-2191) |
| Energy (MJ) | 8.4 ± 1.3  8.2 (7.4-9.2) | 8.9 ± 1.5  8.7 (7.8-9.8) | 8.3 ± 1.2  8.1 (7.4-9.0) | 8.4 ± 1.2  8.2 (7.5-9.2) |
| Protein (g) | 69.3 ± 13.7  68.0 (60.0-77.5) | 75.1 ± 16.0  73.4 (64.0-84.8) | 72.9 ± 14.2  71.6 (63.1-80.7) | 72.6 ± 14.5  71.2 (62.5-81.6) |
| Protein (E%) | 14.1 ± 1.9  14.0 (12.8-15.3) | 14.4 ± 2.1  14.3 (13.0-15.7) | 15.0 ± 1.9  14.9 (13.6-16.1) | 14.7 ± 2.1  14.7 (13.3-16.1) |
| Fat (g) | 77.0 ± 17.1  74.6 (64.7-86.8) | 75.8 ± 19.6  73.4 (61.8-87.0) | 68.0 ± 15.3  66.1 (57.4-76.4) | 62.0 ± 15.1  59.8 (51.4-70.6) |
| Fat (E%) | 35.8 ± 5.6  35.4 (31.8-39.3) | 33.2 ± 6.1  32.7 (28.8-37.1) | 32.0 ± 5.1  31.6 (28.7-35.1) | 28.8 ± 5.4  28.4 (25.2-32.2) |
| Carbohydrate (g) | 239 ± 47.0  234 (205-266) | 264 ± 53.0  259 (225-297) | 246 ± 45.3  241 (215-272) | 272 ± 50.4  268 (236-304) |
| Carbohydrate (E%) | 48.3 ± 5.7  48.4 (44.5-52.1) | 50.4 ± 6.0  50.4 (46.5-54.5) | 50.5 ± 5.8  50.8 (46.6-54.4) | 55.1 ± 6.3  55.3 (51.0-59.1) |
| Alcohol (g) | 3.4 ± 3.1  2.9 (1.5-4.6) | 3.3 ± 3.3  2.7 (1.1-4.5) | 3.4 ± 2.8  3.0 (1.6-4.5) | 3.5 ± 2.9  3.1 (1.6-4.6) |
| Alcohol (E%) | 1.2 ± 1.1  1.0 (0.5-1.6) | 1.1 ± 1.1  0.9 (0.4-1.5) | 1.2 ± 1.0  1.1 (0.5-1.6) | 1.2 ± 1.1  1.0 (0.5-1.6) |
| Sugar (g) | 34.4 ± 15.1  31.9 (23.9-42.2) | 38.9 ± 16.8  35.7 (27.1-47.3) | 33.7 ± 14.1  31.4 (23.9-40.8) | 35.2 ± 13.1  33.3 (26.4-41.7) |
| Sugar (E%) | 7.0 ± 2.7  6.5 (5.0-8.4) | 7.4 ± 2.9  6.9 (5.4-8.8) | 6.9 ± 2.7  6.5 (5.1-8.3) | 7.1 ± 2.4  6.8 (5.5-8.4) |
| Fiber (g) | 20.5 ± 5.5  20.1 (16.6-23.9) | 23.0 ± 6.5  22.5 (18.3-27.0) | 21.9 ± 5.6  21.6 (17.9-25.3) | 30.8 ± 6.9  30.2 (26.2-35.2) |

Abbreviation: E% = percent of energy

^1^ All differences among FPGs significant at *P*≤0.001 (Kruskal-Wallis test)

^2^ Numbers given are mean ± SD; median (25th-75th)

## Additional file 1, Table S2. Macronutrient intake among men classified as Previously Healthy Adequate Reporters (the Västerbotten Intervention Program, 1992-2005)^1, 2^

| Food pattern group  (FPG) (n) | High fat  (1,790) | Tea. soda & cookies  (4,726) | Fruit & vegetables  (2,175) |
| --- | --- | --- | --- |
| Energy (kcal) | 2461 ± 335  2429 (2216-2674) | 2687 ± 406  2649 (2379-2962) | 2566 ± 374  2527 (2289-2809) |
| Energy (MJ) | 10.3 ± 1.4  10.2 (9.3-11.2) | 11.2 ± 1.7  11.1 (10.0-12.4) | 10.7 ± 1.6  10.6 (9.6-11.8) |
| Protein (g) | 82.8 ± 15.9  81.8 (71.8-92.8) | 92.3 ± 19.3  90.2 (78.9-103.5) | 92.7 ± 17.4  91.3 (80.4-103.3) |
| Protein (E%) | 13.7 ± 1.9  13.6 (12.5-14.9) | 14.0 ± 2.0  13.9 (12.7-15.1) | 14.7 ± 1.9  14.7 (13.4-15.9) |
| Fat (g) | 102.8 ± 22.7  99.2 (87.3-115.0) | 109.3 ± 25.1  106.4 (91.4-124.4) | 95.1 ± 21.4  92.5 (80.1-107.9) |
| Fat (E%) | 38.9 ± 6.3  38.5 (34.6-42.5) | 37.9 ± 6.1  37.6 (33.9-41.5) | 34.5 ± 5.6  34.4 (30.7-38.0) |
| Carbohydrate (g) | 285 ± 53.5  280 (247-319) | 315 ± 62.2  310 (271-354) | 317 ± 59.5  311 (275-353) |
| Carbohydrate (E%) | 47.1 ± 6.0  47.2 (43.2-51.0) | 47.7 ± 6.0  47.7 (43.8-51.5) | 50.1 ± 5.8  50.2 (46.4-53.9) |
| Alcohol (g) | 7.1 ± 6.0  5.8 (3.4-9.2) | 7.6 ± 6.7  6.3 (3.4-9.9) | 7.4 ± 5.4  6.5 (3.7-9.8) |
| Alcohol (E%) | 2.0 ± 1.7  1.7 (0.9-2.6) | 2.0 ± 1.8  1.6 (0.9-2.6) | 2.0 ± 1.5  1.8 (1.0-2.7) |
| Sugar (g) | 44.9 ± 20.4  42.5 (30.6-56.2) | 47.3 ± 21.6  43.3 (32.0-58.6) | 44.6 ± 20.2  41.3 (30.3-54.9) |
| Sugar (E%) | 7.4 ± 3.1  7.0 (5.3-9.2) | 7.1 ± 3.0  6.7 (5.0-8.7) | 7.0 ± 3.0  6.6 (4.9-8.6) |
| Fiber (g) | 22.0 ± 6.2  21.5 (17.7-25.9) | 25.0 ± 7.2  24.4 (19.9-29.4) | 26.6 ± 7.5  26.1 (21.0-31.0) |

Abbreviation: E%= percent of energy

^1^ All differences among FPGs significant at *P*≤0.001 (Kruskal-Wallis test)

^2^ Numbers given are mean ± SD; median (25th-75th)

## Additional file 1, Table S3. Micronutrient intake among women classified as Previously Healthy Adequate Reporters, (the Västerbotten Intervention Program, 1992-2005)^1, 2^

| Food pattern group  (FPG) (n) | High fat (3,398) | Tea & ice-cream (3,122) | Coffee & sandwich (2,100) | Fruit & vegetables (756) |
| --- | --- | --- | --- | --- |
| Retinol (mg) | 0.8 ± 0.3  0.7 (0.6-0.9) | 0.8 ± 0.4  0.7 (0.5-1.0) | 0.8 ± 0.3  0.7 (0.6-1.0) | 0.7 ± 0.3  0.6 (0.4-0.8) |
| Carotenoid (mg) | 4.5 ± 3.6  3.5 (1.9-5.9) | 5.7 ± 4.5  4.6 (2.5-8.1) | 4.7 ± 3.7  3.8 (2.0-6.2) | 15.0 ± 9.4  11.9 (8.1-26.4) |
| Vitamin B_2_ (mg) | 1.6 ± 0.4  1.6 (1.3-1.8) | 1.7 ± 0.4  1.7 (1.4-2.0) | 1.7 ± 0.4  1.6 (1.4-1.9) | 1.8 ± 0.4  1.7 (1.5-2.1) |
| Vitamin B_6_ (mg) | 2.1 ± 0.5  2.0 (1.7-2.4) | 2.4 ± 0.6  2.3 (2.0-2.7) | 2.3 ± 0.5  2.2 (1.9-2.6) | 2.8 ± 0.7  2.7 (2.3-3.1) |
| Vitamin B_12_ (µg) | 5.1 ± 1.6  4.9 (4.0-6.0) | 5.4 ± 1.9  5.1 (4.1-6.4) | 5.3 ± 1.7  5.1 (4.1-6.2) | 5.0 ± 1.8  4.8 (3.7-5.9) |
| Vitamin C (mg) | 82 ± 42  73 (53-103) | 102 ± 53  90 (66-124) | 88 ± 44  78 (57-110) | 150 ± 61  138 (106-176) |
| Vitamin D (µg) | 5.5 ± 1.5  5.4 (4.5-6.4) | 5.6 ± 1.8  5.4 (4.4-6.6) | 5.9 ± 1.5  5.7 (4.9-6.7) | 5.2 ± 1.6  5.1 (4.1-6.1) |
| Tokoferol (µg) | 7.0 ± 2.0  6.6 (5.6-7.9) | 7.5 ± 2.1  7.2 (6.0-8.6) | 7.1 ± 1.7  6.9 (5.9-8.0) | 8.7 ± 2.2  8.3 (7.3-9.8) |
| Folate(µg) | 253 ± 65  247 (208-291) | 292 ± 84  282 (233-340) | 271 ± 66  264 (224-310) | 448 ± 125  425 (357-527) |
| Calcium (mg) | 912 ± 285  898 (708-1087) | 975 ± 318  945 (752-1160) | 976 ± 301  945 (767-1154) | 987 ± 309  961 (778-1173) |
| Iron (mg) | 14.3 ± 3.3  13.9 (12.0-16.3) | 15.8 ± 3.8  15.4 (13.1-18.0) | 14.8 ± 3.4  14.3 (12.5-16.7) | 14.8 ± 3.1  14.4 (12.7-16.7) |
| Selen (µg) | 24.1 ± 6.0  23.4 (20.4-27.4) | 26.3 ± 7.8  25.2 (21.4-29.9) | 25.1 ± 6.0  24.3 (21.1-28.2) | 27.6 ± 7.4  26.4 (22.4-31.2) |
| SAFA (mg) | 33.9 ± 8.4  32.7 (28.1-38.4) | 31.8 ± 9.0  30.6 (25.4-36.8) | 27.8 ± 6.6  27.0 (23.3-31.4) | 24.1 ± 6.5  23.3 (19.4-27.9) |
| Cholesterol (g) | 0.24 ± 0.06  0.23 (0.20-0.27) | 0.23 ± 0.07  0.23 (0.19-0.27) | 0.20 ± 0.05  0.20 (0.16-0.23) | 0.18 ± 0.05  0.18 (0.14-0.21) |
| FA 14:0 (g) | 4.2 ± 1.4  3.9 (3.3-4.8) | 3.7 ± 1.4  3.4 (2.7-4.4) | 3.0 ± 0.8  3.0 (2.4-3.5) | 2.6 ± 1.0  2.5 (1.9-3.1) |
| FA 16:0 (g) | 16.8 ± 3.8  16.2 (14.0-18.9) | 16.1 ± 4.2  15.6 (13.1-18.5) | 14.1 ± 3.1  13.8 (12.0-15.9) | 12.9 ± 3.1  12.5 (10.7-14.8) |
| FA 18:2 (g) | 7.5 ± 2.8  6.9 (5.8-8.5) | 8.8 ± 3.9  7.8 (6.3-10.1) | 7.7 ± 2.9  7.0 (5.9-8.6) | 9.3 ± 4.8  7.9 (6.1-10.6) |
| FA 18:3 (g) | 1.6 ± 0.5  1.5 (1.3-1.9) | 1.8 ± 0.7  1.6 (1.3-2.0) | 1.6 ± 0.5  1.5 (1.3-1.8) | 1.8 ± 0.8  1.6 (1.3-2.1) |
| FA 20:5 (g) | 0.07 ± 0.05  0.06 (0.05-0.09) | 0.08 ± 0.05  0.06 (0.05-0.09) | 0.07 ± 0.04  0.06 (0.05-0.09) | 0.08 ± 0.05  0.07 (0.05-0.09) |
| FA 22:6 (g) | 0.15 ± 0.10  0.13 (0.09-0.18) | 0.15 ± 0.11  0.13 (0.10-0.18) | 0.14 ± 0.08  0.13 (0.09-0.18) | 0.15 ± 0.09  0.13 (0.10-0.18) |

Abbreviations: SAFA = saturated fatty acids; FA = fatty acids

^1^ All differences among FPGs significant at *P*≤0.001 (Kruskal-Wallis test), except FA 20:5, *P*=0.032 and FA 22:6, *P*=0.007

^2^ Numbers given are mean ± SD; median (25th-75th)

## Additional file 1, Table S4. Micronutrient intake among men classified as Previously Healthy Adequate Reporters (the Västerbotten Intervention Program, 1992-2005)^1, 2^

| Food pattern group  (FPG) (n) | High fat  (1,790) | Tea. soda & cookies  (4,726) | Fruit & vegetables  (2,175) |
| --- | --- | --- | --- |
| Retinol (mg) | 1.0 ± 0.4  0.9 (0.7-1.2) | 1.0 ± 0.5  0.9 (0.7-1.2) | 1.1 ± 0.5  1.0 (0.8-1.3) |
| Carotenoid (mg) | 2.6 ± 2.4  1.8 (1.2-3.3) | 3.6 ± 3.4  2.5 (1.5-4.5) | 4.1 ± 4.0  2.7 (1.6-5.0) |
| Vitamin B_2_ (mg) | 1.9 ± 0.5  1.8 (1.5-2.2) | 2.1 ± 0.6  2.0 (1.7-2.4) | 2.1 ± 0.5  2.1 (1.7-2.4) |
| Vitamin B_6_ (mg) | 2.3 ± 0.6  2.3 (1.9-2.6) | 2.6 ± 0.7  2.5 (2.1-3.0) | 2.8 ± 0.6  2.7 (2.3-3.1) |
| Vitamin B_12_ (µg) | 6.7 ± 2.4  6.3 (5.0-7.9) | 7.3 ± 2.8  6.8 (5.4-8.6) | 7.2 ± 2.7  6.8 (5.4-8.5) |
| Vitamin C (mg) | 82 ± 44  73 (52-103) | 103 ± 53  94 (66-126) | 105 ± 55  94 (66-127) |
| Vitamin D (µg) | 7.5 ± 2.1  7.3 (6.1-8.6) | 7.7 ± 2.4  7.5 (6.1-9.0) | 8.3 ± 2.2  8.0 (6.8-9.5) |
| Tokoferol (µg) | 8.2 ± 2.7  7.5 (6.5-9.1) | 8.8 ± 2.6  8.3 (7.1-10.0) | 9.0 ± 2.2  8.6 (7.4-10.2) |
| Folate(µg) | 263 ± 65  258 (217-300) | 300 ± 79  290 (246-342) | 316 ± 84  303 (256-361) |
| Calcium (mg) | 1057 ± 350  1035 (796-1279) | 1142 ± 388  1101 (867-1365) | 1179 ± 369  1142 (920-1415) |
| Iron (mg) | 16.8 ± 3.7  16.4 (14.3-19.0) | 19.0 ± 4.2  18.7 (16.0-21.5) | 19.0 ± 4.0  18.7 ± 16.1-21.4) |
| Selen (µg) | 28.2 ± 7.0  27.6 (23.5-31.9) | 31.4 ± 8.7  30.2 (25.6-35.7) | 31.3 ± 7.7  30.3 (25.9-35.6) |
| SAFA (mg) | 45.5 ± 11.8  43.4 (37.9-51.2) | 47.7 ± 12.6  46.1 (39.0-54.7) | 38.2 ± 9.3  37.2 (31.8-43.5) |
| Cholesterol (g) | 0.30 ± 0.07  0.30 (0.25-0.35) | 0.32 ± 0.08  0.32 (0.27-0.37) | 0.26 ± 0.07  0.25 (0.21-0.30) |
| FA 14:0 (g) | 5.5 ± 1.8  5.2 (4.3-6.3) | 5.6 ± 1.9  5.4 (4.4-6.6) | 4.1 ± 1.3  4.0 (3.2-4.8) |
| FA 16:0 (g) | 22.3 ± 5.4  21.6 (18.7-25.2) | 23.5 ± 5.9  22.8 (19.4-27.1) | 19.5 ± 4.6)  19.1 (16.2-22.3) |
| FA 18:2 (g) | 9.9 ± 3.6  9.1 (7.7-11.2) | 11.4 ± 4.7  10.3 (8.4-13.0) | 11.6 ± 5.0  10.4 (8.6-13.0) |
| FA 18:3 (g) | 2.2 ± 0.7  2.1 (1.7-2.6) | 2.4 ± 0.8  2.2 (1.8-2.8) | 2.4 ± 0.8  2.2 (1.8-2.8) |
| FA 20:5 (g) | 0.09 ± 0.06  0.08 (0.05-0.11) | 0.10 ± 0.07  0.09 (0.06-0.12) | 0.10 ± 0.06  0.08 (0.06-0.12) |
| FA 22:6 (g) | 0.18 ± 0.12  0.16 (0.11-0.22) | 0.20 ± 0.14  0.17 (0.12-0.24) | 0.19 ± 0.11  0.17 (0.12-0.23) |

Abbreviations: SAFA = saturated fatty acids; FA = fatty acids

^1^ All differences among FPGs significant at *P*≤0.001 (Kruskal-Wallis test)

^2^ Mean ± SD; median (25th-75th)
